# Inflammation, HIF-1, and the Epigenetics That Follows

**DOI:** 10.1155/2010/263914

**Published:** 2010-12-19

**Authors:** Claudio Brigati, Barbara Banelli, Angela di Vinci, Ida Casciano, Giorgio Allemanni, Alessandra Forlani, Luana Borzì, Massimo Romani

**Affiliations:** Tumor Genetics Laboratory, National Institute for Cancer Research, Largo Benzi, 10, 16132 Genova, Italy

## Abstract

We summarize recent findings linking inflammatory hypoxia to chromatin modifications, in particular to repressive histone signatures. We focus on the role of Hypoxia-Induced Factor-1 in promoting the activity of specific histone demethylases thus deeply modifying chromatin configuration. The consequences of these changes are depicted in terms of gene expression and cellular phenotypes. We finally integrate available data to introduce novel speculations on the relationship between inflammation, histones, and DNA function and integrity.

## 1. Introduction

Epigenetic control is a motor of gene regulation. In a broad sense, it can be viewed as a “bridge” between genotype and phenotype, as being capable of changing the function of a given locus without modification of the underlying sequence. This occurs because both DNA and histones can be chemically modified in the cell without changing the base composition, and these modifications are read by the transcriptional apparatus as a on/off signal. DNA can be methylated, when needed, mainly on promoter cytosines, whereas histones can be modified on lysines, and less frequently on arginines, by methylation and other modifications, creating a template for heritable changes in gene expression, the epigenome. In evolutionary terms, the evolving epigenome is probably increasing the variability and adaptation of species, as recently shown for Schistosoma [1]. Moreover, epigenetic modifications are now viewed both as localized locus-specific changes acting at the single-gene level and as a global change, capable of reprogramming vast areas in genomes if needed. The epigenome is the result of a developmental genetic asset and of the lifetime action of environment. The main environmental factors able to cause this extended effect are not well characterized, but metabolism is emerging as an important candidate. Indeed, the concurrence of metabolic events and epigenetic changes in gene expression has a long, often anecdotal record in science. Metabolism seems to be a prime force behind epigenetic configuration and activity, and in this paper we focus on recent molecular findings linking inflammatory hypoxia to epigenome configurations; but it should be pointed out that, reciprocally, epigenetics may regulate cardinal aspects of cell metabolism as shown by strong epidemiological data [2] even to the point of causing transgenerational effects (see below).

## 2. Epigenetics: Histones under the SpotlightAgain!

More than 40 years ago, Vincent Allfrey proposed that histone modifications could contribute significantly to the regulation of gene expression [3] but only recently progress has been made in elucidating in great detail the chemical nature of these changes and their relationship with metabolism [4–7]. Now it is known that besides the well-established HAT/HDAC (histone acetylase/deacetylase) equilibrium, in which acetylation or deacetylation is strictly correlated to gene activation or repression, respectively, histones are known to undergo dozens of other specific modifications, contributing to the finely tuned control of gene expression. One such modification is the enzymatic control of histone 3 methylation status at lysine residues; this is viewed as a central event, linked to a number of cellular processes including DNA repair, replication, transcriptional activation, and repression [4]. 

Histone methylation was once considered enzymatically irreversible until a histone lysine demethylase (LSD1) was identified [8]. Subsequently, a large family of jumonji family (JmjC) domain-containing histone lysine demethylases has been isolated and their mechanism of action elucidated [9, 10]. The existence of inducible enzymes erasing a histone methylation mark prompted the concept of bistable states. Bi-stable systems can exist in usually two alternative states under the same conditions. One interesting property is that one of these acquired states can be maintained even after an external stimulus that initiated the switch is extinguished, a property called hysteresis [11]. Events such as X chromosomal inactivation or Hox gene expression could be examples of such regulation. Another is presumably hyperglycemic memory, a metabolic situation in which the deleterious effects of the initial insult are remembered by the organism despite the return to normal glucose levels (see below).

## 3. Inflammatory Hypoxia Induces HIF-1

In multicellular organisms, oxygen is supplied essentially by blood circulation, which brings molecular O_2_ to long distances and wide areas via an intricate network of vessels, culminating in capillaries. Low oxygenation (hypoxia) occurs when cells are at a distance greater then roughly 200 nm from these capillaries, while, within this distance, oxygen can freely diffuse from cell to cell [12]. Hypoxia is a common experience in a life of an animal and a frequent event in inflammation: in acute inflammations (such as many infections), it is driven mainly—but not exclusively—by factors such as elevated O_2_ consuption by the pathogen or first-aid immune cells, or transient ischemia due to vasoconstriction or compression by local edema; ischemia may be a more relevant factor in subacute or chronic inflammations, due for instance to vessel clogging by incoming cellular infiltrate; moreover, some anatomical districts may be more prone to undergo inflammatory hypoxia [13].

Hypoxia, including that induced in inflammatory processes, induces Hypoxia-Inducible Factor (HIF-1)*α*, which binds to and activates regulatory regions of target genes [14]. To do so, it must first bind its partner, HIF-1*β*, and this interaction is controlled by oxygen levels. When oxygen is abundant, there is little HIF-1*α*, it is destroyed under the direction of the von Hippel-Lindau (VHL) protein—and what is left cannot bind HIF-1*β*. At low oxygen levels, these restraints are lifted [15].

HIF-1 is pleiotropic: targets include factors involved in metabolism and angiogenesis, like the inducible form of nitric oxide (NO) synthase (iNOS), vascular endothelial growth factor (VEGF), glucose transporter-1, and several glycolytic enzymes. Accordingly, studies utilizing knock-out mice have shown that HIF-1 deletion is lethal during midterm of embryonic development resulting in major defects particularly in the vascular system [16]. 

**Figure 1:**
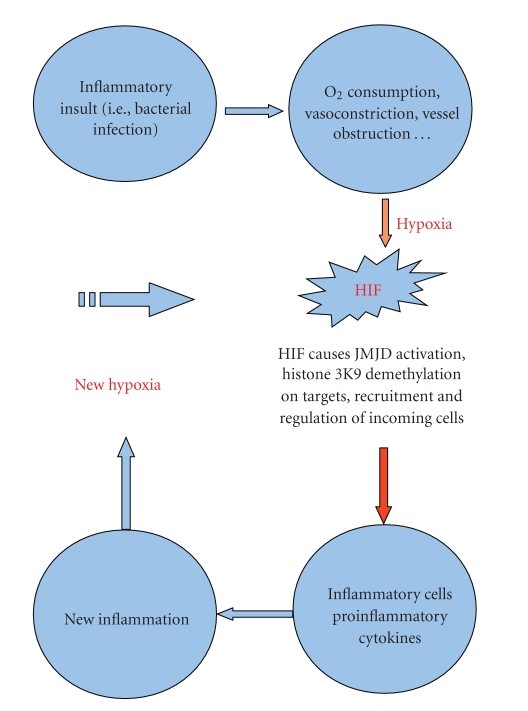
A vicious circuit could be established when HIF-1 activates, on recruited inflammatory cells, specific cytokines, such as IL-10, TNF, HIF1 (on macrophages in particular), or IL-4, IL13 (from mastocytes, eosinophils, or other cells). This event would create new inflammation followed by new hypoxia again.

Inflammation is usually self-contained both in temporal and structural terms, but one complicating aspect is that a vicious circuit (Figure 1) may potentially take place in which hypoxia itself is bringing about new inflammation, essentially by HIF's capacity of calling and regulating the cellular infiltrate; of cardinal importance among incoming cells are macrophages, which are capable of responding promptly to the stimulus (i.e., infections) and inducing a wide array of proinflammatory mediators such as MIF, TNF, IL-6, IL-10, and HIF-1 itself again [17–20].

## 4. HIF-1 Acting on Specific HistoneDemethylases: a Dangerous Liaison?

In addition to the above-mentioned HIF's direct transactivation of target genes, recent work pointed to HIF-1 as being capable of “indirect”, albeit dramatic modification of chromatin structure, particularly in terms of histone code configuration.

What is the link between HIF-1 and histone modifications? First, in recent years, evidence has pointed towards HIF-1 capability of HDAC recruitment and regulation [20, 21]. Then, and importantly, HIF-1 has been found to bind specific sites on the promoter of the histone 3 lysine 9 demethylases thereby inducing their expression. In particular, it induces JMJD1A and JMJD2A that are devoted to removal of dimethyl marks on histone 3 lysine 9 (H3K9me2) JMJD2B [22, 23] which removes trimethyl marks (H3K9me3) and more weakly JMJD2C which converts H3K9me3 to me2 [24].

Before speculating on the consequences of JMJD activation in inflammatory hypoxia, we should recollect what is known on the putative role of H3K9 modification in cell physiology.

Currently, H3K9 methylation has been linked to the determination and building of repressive heterochromatin; for instance, it is established that H3K9 di- and trimethylation creates the binding site for HP1, a conserved heterochromatin protein that mediates gene silencing, heterochromatin compaction, and late replication [25]. H3K9 di-trimethylation is enriched in pericentromeric heterochromatin, and it is dependent on the Suv39H HMTase activity [26]. However, pericentromeric chromatin may also be under complex control; for instance, ncRNAs may play a role, as loss of RNAi leads to loss of silencing and H3K9 dimethylation from reporter genes embedded within centromeric repeats, at least in fission yeast [27]. 

In Drosophila, it has been shown that HP1 is required for the normal organization of the nucleolus and heterochromatic DNA repeats, and that H3K9 methylation protects these repeats from being excised from the genome by the enzyme Ligase 4 [28]. Overall, one strong notion is that because of H3K9 methylation, the binding of HP1 and the presence of a compacted chromatin may create a protective barrier against DNA damage during replication and may reduce the sensitivity to DNA disrupting agents [28].

On a speculative basis, the removal of H3K9 mark and the lifting of this protection in inflammation could expose the cell to the occurrence of cancer initiating events (in the form of chromosomal instability). Thus, besides the long recognized role of (chronic) inflammation as instrumental to cancer progression [29, 30], new data linking inflammation to initiation can be added to this emerging field [31, 32].

Importantly, the notions discussed in the present paper indicate one possible (epigenetic) mechanism whereby the following may occur HIF-1 mediated induction of histone demethylases, removal of H3K9 methyl marks, loosening of chromatin compaction, exposure of DNA to instability, or abnormal transcription of otherwise silent genes.

The perspective linking inflammation to cancer initiation is obviously a worrisome one, and it is likely that safeguard mechanisms, such as p53 activation, would be called often for extra work to protect tissues from dangerous karyotypes arising [33].

## 5. A Cellular Scenario Arising

Which transcriptome scenario—hence cellular phenotype—should be expect within inflammatory hypoxic areas? In a simplified (and speculative) view, together with the large panel of targets known to be affected by HIF-1 as a transactivator, we could witness a whole set of genes being rapidly derepressed as a result of increased JMJD function, with unpredictable consequences on cell life. Moreover, in a genomic “topographical” sense, the extent of this derepression could be significant if long distance spreading of the newly acquired modification would take place [34]. Given the known cross-talk between histone and DNA modifications [35], it would also be of interest to study what influence the subtraction of such histone repressive mark may exert on loci bearing repressive methylation on their promoter DNA, as in the PGC-1*α* gene in cells treated by the proinflammatory fatty acid palmitate [36]. Alteration of the equilibrium between opposing chromatin-modifying activities (histone code) might also allow the system to jump from one state to another (bistability). It remains to be seen if such waves of epigenetic alteration could reach catastrophic dimensions, like in nuclear reprogramming [37]. Likely, again, under usual conditions adaptation could restrain the number of targets or limit spreading in order to ensure the proper evolution of an inflammatory insult.

Cellular consequences could than range from minor to significant, depending on the cell maturation level, the time frame of activation, and the relevance of some target genes. In an inflammatory context, metaplasia of differentiated cells has been postulated as one possible outcome [38]. Hypoxia (via JMJD1A and JMJD2c function) has been implicated in regulating the properties of stem cells [39, 40]; exit from stemness would be favoured by the deposition in key genes of the H3K9 di-trimethylation marks. Thus, one could speculate that JMJD induction would erase the marks, counteract the silencing, and presumably help maintaining the self-renewal state even when not appropriate. In this case, we could envisage hypoxia as a potential reactivator of genes that are regulated similarly to Polycomb Group (PcG) targets, known to be sensitive to H3K27 demethylases [38, 41]. This event would be a potential oncogenic occurrence: histone repressive marks are thought to keep certain loci on a hold until a given level of expression is needed, thus their writing and reading are a developmentally regulated process that requires tight control throughout life. Although timely reactivation of some targets would be beneficial to differentiation and tissue repair during inflammation, other genes must be kept silent at that time point to maintain the stem cell pool. The equilibrium between these compartments is likely to be disrupted in tumours, and this allows us to look at a cellular theory of cancer with new eyes after so long has passed since the Virchow hypothesis (1849). It is therefore central to complete the annotation of the relevant JMJD demethylase targets in a given tissue and in a given cell type exposed to hypoxia. Technology is currently available to this purpose and some results relative to kidney and colon have already been published [42].

## 6. Conclusions

The discovery of a relationship between environmental clues and H3 lysine demethylases sheds new light on the intricacies of gene expression and advances our understanding on the epigenetics of inflammation. 

We should here stress that, although in this paper we have focused mainly on the reduction of oxygen tension, it is clear that, in inflammation, a whole array of molecular events could concur to alter the histone code, with important consequences on cell life. For instance, it was found that, in hyperglycemic memory,NF*κ*Bhigh transcriptional activity is due to enzymatic erasing of H3K9 methyl marks from its promoter [43, 44]. This finding discloses a new perspective on the blueprint left by our alimentary (and possibly other) habits and emphasizes the link between metabolism and inflammation (henceNF*κ*Bactivity) in shaping our evolving epigenome [45, 46].

Moreover, recent findings suggest that even trans-generational differences can be due to environmental clues (such as exposure to chemical compounds or mother's diet) acting in the temporal windows of gametogenesis and embryo implantation, during which massive epigenetic reprogramming takes place [47]. The impact of inflammatory hypoxia occurring within these developmental time frames has not been evaluated yet but it could now deserve attention.

Together, these findings have broad implications for how we envisage the mechanisms underlying epigenetics and the every day physiology and offer us a mechanistic glimpse into the exciting world of dynamic chromatin.

## Abbreviations

HAT/HDAC: Histone acetylatilase/deacetylase
JMJD: Jumonji-domain-containing histone demethylases
H3K9me2,H3K9me3: Di, trimethylated histone3-lysine9
ncRNA: Noncoding RNA
RNAi: RNA interference.
